# Differential photoacoustic-stimulated Raman spectroscopy (DPA-SRS) for high-sensitivity hydrogen detection

**DOI:** 10.1016/j.pacs.2026.100814

**Published:** 2026-02-25

**Authors:** Xin Yu, Zhengang Li, Jiaxiang Liu, Haichun Xu, Junfang Miao, Canlong Wang, Yongqing Fang, Ying Pan, Yonghua Fang

**Affiliations:** a Key Laboratory of Environmental Optics and Technology, Anhui Institute of Optics and Fine Mechanics, Hefei Institutes of Physical Science, Chinese Academy of Sciences, Hefei 230031, China; b University of Science and Technology of China, Hefei 230026, China

**Keywords:** Hydrogen, Photoacoustic spectroscopy, Differential photoacoustic-stimulated Raman, Four-wave mixing, Weak Signal Processing

## Abstract

To detect non-polar, infrared-inactive hydrogen, a Differential Photoacoustic-Stimulated Raman Spectroscopy (DPA-SRS) method is proposed. Utilizing the SRS process, a portion of the pump light is converted into intense Stokes light corresponding to the hydrogen Raman shift, eliminating complex dual-laser configurations. The nonlinear thermoacoustic effect is excited by this dual-color light field, endowing Photoacoustic Spectroscopy with the capability for hydrogen fingerprint identification. Raman cell pressure was optimized to achieve a synergistic enhancement of the Stokes conversion efficiency and the Four-Wave Mixing effect. Furthermore, an acoustic mode-optimized differential H-type resonant photoacoustic cell was designed, which effectively enhances anti-interference capability through the differential detection mechanism. Distinct from traditional lock-in amplification methods, a time-frequency transformation algorithm was employed to precisely extract the frequency-domain photoacoustic signal from the broadband time-domain acoustic signal. Experimental results demonstrate that the DPA-SRS system exhibits excellent linearity and achieves a Limit of Detection of 0.65 ppm under atmospheric conditions.

## 1 Introduction

Hydrogen (H_2_), recognized as a highly promising clean energy carrier, has found extensive applications in critical sectors such as aerospace, fuel cells, the petrochemical industry, and semiconductor manufacturing. However, characterized by its small molecular radius, high permeability, colorless and odorless nature, and extreme flammability and explosiveness [1] (with an explosive limit in air ranging from 4% to 75%), hydrogen poses significant risks. Any leakage occurring during production, storage, transportation, or application processes may precipitate severe safety accidents [2]. Therefore, the development of high-sensitivity, real-time hydrogen monitoring technologies is not only an urgent requirement for safeguarding industrial production and protecting human life and property but also serves as a core technical pillar supporting the large-scale and safe deployment of the hydrogen economy.

Current industrial hydrogen detection primarily relies on gas chromatography, electrochemical sensors, and thermal conductivity sensors [3], [4], [5]. However, limited by slow response times, susceptibility to environmental cross-interference (e.g., CO interference), and sensor poisoning, these traditional methods struggle to achieve long-term, stable trace monitoring in the complex background.

In the field of optical detection, infrared absorption techniques such as photoacoustic spectroscopy (PAS) [6], [7], [8], [9], [10], [11], [12], [13], [14], tunable diode laser absorption spectroscopy (TDLAS) [15] and light-induced thermoelastic spectroscopy (LITES) [16], [17], [18], [19], [20], [21], [22], [23] are known for their high sensitivity. However, since the hydrogen molecule lacks a permanent dipole moment and is infrared-inactive [24], [25], direct detection based on absorption spectroscopy principles proves difficult. Building upon traditional PAS technology, researchers have proposed an indirect photoacoustic spectroscopy technique that retrieves hydrogen concentration by analyzing variations in the resonance frequency of the photoacoustic cell. When a certain concentration of hydrogen is present in a photoacoustic cell, the speed of sound in the gas mixture changes significantly, thereby affecting the photoacoustic resonance frequency. Measuring this frequency shift allows for the retrieval of hydrogen concentration.

Based on this principle, Wang et al. [26], [27] designed two detection systems utilizing the sensitivity of resonant photoacoustic cells to frequency shifts. Using a loudspeaker as an acoustic excitation source, they analyzed the resonance frequency deviation of the cell filled with varying concentrations of the target gas to determine H_2_ and O_2_ levels. Similarly, Ye et al. [28] developed a photoacoustic hydrogen detection device using sound waves released by water vapor as the excitation source, achieving simultaneous detection of H_2_ and H_2_O with a detection limit of approximately 138.69 ppm. While these methods allow for hydrogen detection to some extent, they demand extremely high machining precision for the photoacoustic cell, as minute deviations can lead to resonance frequency instability. Furthermore, they are significantly affected by environmental factors such as temperature and humidity. Additionally, detecting unknown multi-component gases requires multiple auxiliary light sources, greatly increasing system complexity.

Additionally, Raman spectroscopy, as a scattering spectroscopic technique [29], [30], [31], possesses unique gas fingerprinting capabilities, making it inherently suitable for the detection of homonuclear diatomic molecules such as H_2_. However, the efficiency of spontaneous Raman scattering is extremely low (the scattering cross-section is typically 3–6 orders of magnitude lower than that of Rayleigh scattering) [32], [33], resulting in extremely weak signals that severely limit its potential for trace gas detection. To achieve low-concentration hydrogen detection, researchers have developed enhanced Raman spectroscopy techniques capable of signal amplification. Taylor et al. [34] electronically stabilized a 1 W laser within a resonant cavity to achieve an intracavity power of 50 W, obtaining a hydrogen detection limit of 10 ppm. Hippler et al. [35] reported a cavity-enhanced Raman spectroscopy setup based on an optical feedback diode laser. By using a 10 mW diode laser, they calculated a detection limit of 140 ppm. Furthermore, our research group [36] has investigated cavity enhancement by designing a parabolic mirror cavity, which improved the collection efficiency of Raman scattering signals while achieving multiple excitations of the gas, realizing effective detection of H_2_ at 1 atm.

To overcome the limitations of single-spectrum techniques, Barrett et al. [37] developed a combined detection method known as Photoacoustic-Raman Spectroscopy (PARS), which integrates the high sensitivity of photoacoustic spectroscopy with the specific selectivity of Raman spectroscopy. The principle involves utilizing a fundamental pump beam and a probe beam (corresponding to the Stokes frequency shift of hydrogen) as coherent excitation sources to induce stimulated Raman transitions in hydrogen molecules. By capturing the thermal energy released from vibrationally excited states via non-radiative collisional (*V*-*T*) relaxation processes, the inherently weak Raman scattering process is converted into a macroscopic, measurable acoustic signal. This “light-heat-sound” conversion mechanism significantly amplifies the response gain of the Raman signal, offering the potential for high-sensitivity detection of trace hydrogen. However, in conventional PARS systems, the Stokes beam is typically generated by dye lasers, which are bulky and expensive, thereby limiting their practical applications. Concurrently, advancements in Stimulated Raman Scattering (SRS) technology have demonstrated that efficient frequency conversion of pump light can be achieved using a single pump laser combined with a Raman shifter [38]. This offers a novel approach for optimizing PARS systems.

To this end, a high-sensitivity Differential Photoacoustic-Stimulated Raman Spectroscopy (DPA-SRS) detection method based on Stimulated Raman Scattering (SRS) enhancement is proposed. First, the generation mechanism of PARS signals is systematically analyzed to elucidate the dependence of the photoacoustic signal intensity on the pump and Stokes beam intensities. Second, by employing a single laser coupled with a high-pressure Raman shifter, a portion of the pump light is self-adaptively converted into Stokes light corresponding to the hydrogen Raman shift, thereby establishing a self-aligned dual-wavelength excitation source. Through the optimization of the shifter pressure to 13 atm, a synergistic enhancement of the Stokes conversion rate and the Four-Wave Mixing (FWM) effect is achieved. Finally, to mitigate the noise fluctuations introduced by the high-power pulsed laser, a custom-designed differential H-type photoacoustic cell is integrated with weak signal processing algorithms, successfully lowering the Limit of Detection (LOD) for hydrogen to the sub-ppm level.

## 2 Theoretical analysis of PARS

**Fig. 1:**
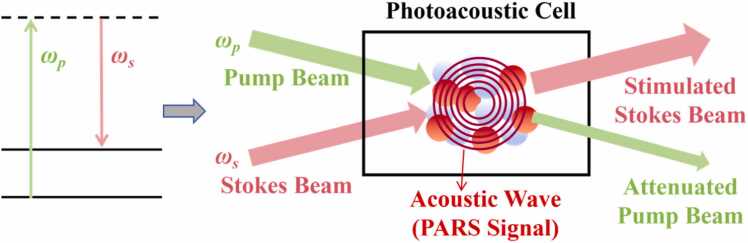
Mechanism of PARS signal generation.

The generation of PARS signals is fundamentally based on the stimulated Raman transition process, which involves the nonlinear interaction between incident pump photons and the vibrational energy levels of the molecular medium, as illustrated in Fig. 1. When the pump beam $\omega_{p}$ and the Stokes beam $\omega_{s}$ interact simultaneously with the gaseous medium, the molecules absorb the energy ($\Delta E=\hbar (\omega_{p}-\omega_{s})$) by coherent resonance, transitioning from the ground state to a vibrationally excited state. Macroscopically, this manifests as a stimulated gain in the Stokes beam. Subsequently, the excited molecules dissipate their vibrational energy into local thermal energy via non-radiative collisional relaxation processes. This results in an instantaneous thermal expansion of the gas within the detection region, thereby exciting an acoustic wave. This pressure variation induced by the stimulated Raman gain constitutes the raw photoacoustic signal in PARS technology.

According to the theory of stimulated Raman scattering, the intensity of the Stokes beam $I_{s}(z)$ after propagating a distance *z* (cm) through the medium follows an exponential growth law [39]:

(1) $$I_{s}(z)\;=\;I_{s}(0)e^{g_{s}z}$$

where $I_{s}(0)$ is the initial intensity (${W/\mathrm{cm}}^{2}$) of the Stokes beam, and $g_{s}$ denotes the Raman gain coefficient (${\mathrm{cm}}^{-1}$) per unit length within the medium. Under weak gain conditions ($g_{s}z\ll 1$), the exponential term can be approximated using a first-order Taylor expansion (i.e., $e^{x}\;\approx \;1+x$). Consequently, Eq. (1) simplifies to:

(2) $$I_{s}(z)\;\approx {\;I}_{s}(0)(1+g_{s}z)$$

Thus, the increment in Stokes beam intensity can be expressed as:

(3) $$\text{Δ}I_{s}\;=\;I_{s}(z)-I_{s}(0)\;\approx \;I_{s}(0)g_{s}z$$

The gain coefficient $g_{s}$ at the peak of the Raman transition can be expressed in terms of the spontaneous Raman scattering cross-section and the pump intensity [37]:

(4) $$g_{s}\;=\;\frac{16\pi^{3}Nc^{2}}{\text{h}n_{s}n_{p}\omega_{s}^{3}\text{Γ}}\left(\frac{d\sigma }{d\text{Ω}}\right)I_{p}$$

where N is the molecular number density (${\mathrm{cm}}^{-3}$), h is Planck constant (J∙s), c is the speed of light (m/s), $n_{s}$ and $n_{p}$ are the refractive indices at the Stokes and refractive index (Dimensionless), respectively, $\omega_{s}$ is the Stokes angular frequency (rad/s), Γ represents the Raman linewidth (rad/s), $I_{p}$ is the pump laser intensity (${W/\mathrm{cm}}^{2}$), and dσ/dΩ is the spontaneous Raman scattering cross-section (${\mathrm{cm}}^{2}/\mathrm{sr}$). Letting the physical constants in Eq. (4) be represented by K (m/W), the effective gain coefficient ${\;g}_{s}$ (${\mathrm{cm}}^{-1}$), which depends on the pump intensity, can be simplified as:

(5) $${\;g}_{s}=K∙I_{p}$$

Substituting this into the intensity increment Eq. (3), the change in Stokes beam intensity depends simultaneously on the intensities of both beams:

(6) $$\text{Δ}I_{s}\;\approx {\;I}_{s}(0)KI_{p}z\;\propto \;I_{s}(0)I_{p}$$

In the stimulated Raman process, energy exchange within the optical field adheres to photon number conservation. For every Stokes photon generated, one pump photon is consumed, while simultaneously exciting one molecule from the ground state to a vibrationally excited state (i.e., Energy level v=1) [40]. Therefore, the total number of excited molecules $\text{Δ}N_{1}$ equals the number of added Stokes photons. Assuming a laser pulse interaction time T (s), beam cross-sectional area $A_{s}$ (${\mathrm{cm}}^{2}$), and single Stokes photon energy $\text{ℏ}\omega_{s}$ (J), the number of added photons is the total energy gain divided by the energy of a single Stokes photon:

(7) $$\text{Δ}N_{p\text{h}\mathrm{otons}}=\frac{\text{Δ}I_{s}A_{s}T}{\text{ℏ}\omega_{s}}$$

Since $\text{Δ}N_{1}=\text{Δ}N_{p\text{h}\mathrm{otons}}$, substituting Eq. (3) into the above equation yields the number of excited molecules [41]:

(8) $$\text{Δ}N_{1}=\frac{I_{s}(0)g_{s}zA_{s}T}{\text{ℏ}\omega_{s}}$$

Assuming the excited molecules return to the ground state primarily via rapid non-radiative Vibration-Translation (*V*-*T*) relaxation (ignoring fluorescence emission), the vibrational energy $\text{ℏ}\omega_{0}$ (i.e., $\text{ℏ}(\omega_{p}-\omega_{s})$) is released as translational kinetic energy (heat (J)). The total thermal energy ΔU (J) released into the gas due to non-radiative relaxation is expressed as:

(9) $$\text{Δ}U\;=\text{ Δ}N_{1}∙(\text{ℏ}\omega_{0})$$

Substituting expression (8) yields:

(10) $$\text{Δ}U\;=\;\left[\frac{I_{s}(0)g_{s}zA_{s}T}{\text{ℏ}\omega_{s}}\right]\text{ℏ}\omega_{0}\;=\;{\;I}_{s}(0)g_{s}zA_{s}T\left(\frac{\omega_{0}}{\omega_{s}}\right)$$

According to the ideal gas law, the increase in thermal energy Δp within the closed photoacoustic cell volume V ($m^{3}$) leads to a pressure change Δp (i.e., the PARS signal (Pa)). For an ideal gas [42], the relationship is:

(11) $$\text{Δ}p\;=\;(\gamma -1)\frac{\text{Δ}U}{V}$$

where γ is the adiabatic index (specific heat ratio (Dimensionless)). Substituting (5), (10) into the pressure formula (11), the final expression for the PARS signal intensity is obtained:

(12) $$\text{Δ}p\;=\;\frac{(\gamma -1)A_{s}\mathrm{Tz}}{V}\left(\frac{\omega_{0}}{\omega_{s}}\right)∙I_{s}(0)∙(KI_{p})$$

Upon rearrangement, the constant terms and intensity terms can be clearly separated:

(13) $$S_{\mathrm{PARS}}\;\propto \text{ Δ}p\;=\;\left[\frac{(\gamma -1)KA_{s}\mathrm{Tz}\omega_{0}}{V\omega_{s}}\right]∙I_{p}∙I_{s}(0)$$

The International System of Units (SI) definitions for the relevant physical quantities are as follows: photoacoustic pressure Δp in Pa, pump light intensity $I_{p}$ and initial Stokes light intensity $I_{s}(0)$ in ${\text{W}/\text{cm}}^{2}$, interaction time T in s, interaction length z in cm, beam cross-sectional area $A_{s}$ in ${\text{m}}^{2}$, and the effective volume of the photoacoustic cell V in ${\text{m}}^{3}$. To verify the rigor of the theoretical model, a dimensional analysis was performed on (4), (13). Based on the fundamental dimensional decomposition within the SI system, the dimension of the Raman gain coefficient $g_{s}$ corresponds to the reciprocal of length ($\left[L^{-1}\right]$), which is strictly consistent with the physical definition of the exponential gain law $I_{s}(z)=I_{s}(0)\exp(g_{s}z)$. Accordingly, the energy density term (${\text{J}/\text{m}}^{3}$) on the right side of Eq. (13) is dimensionally unified with the acoustic pressure unit (Pa) on the left side.

Additionally, From Eq. (13), it is evident that the PARS signal amplitude $S_{\mathrm{PARS}}\;$is directly proportional to the product of the pump beam intensity $I_{p}$ and the initial Stokes beam intensity $I_{s}(0)$. This indicates that for a determined photoacoustic cell structure, maximizing the product of the power densities of the two excitation beams is key to enhancing detection sensitivity.

## 3 Experiments and analysis

### 3.1 Experimental setup

**Fig. 2:**
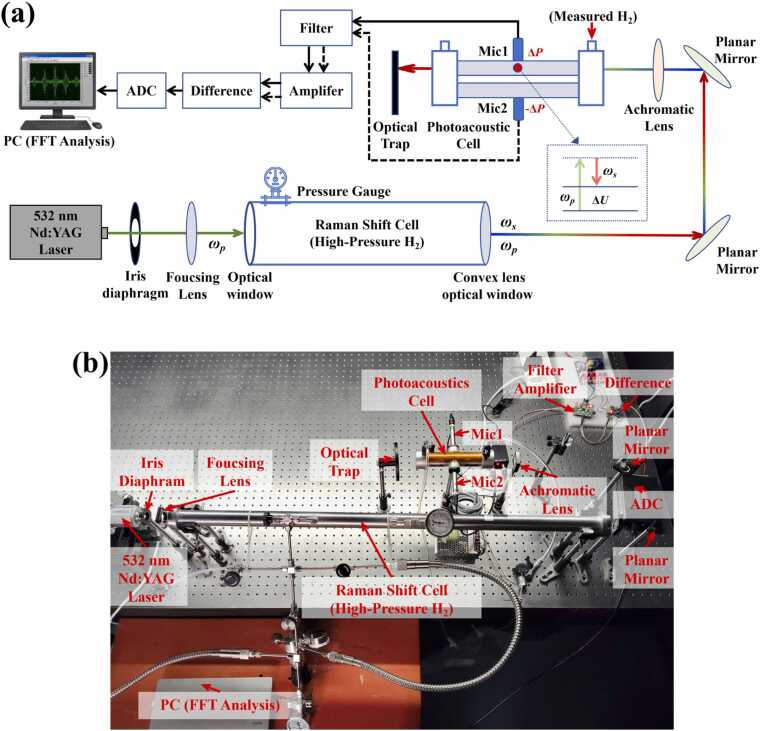
DPA-SRS hydrogen detection system. (a) Schematic diagram; (b) Photograph of the experimental setup.

Building upon the aforementioned theoretical analysis, a high-sensitivity hydrogen detection system utilizing SRS-enhanced DPA-SRS was established. The schematic diagram and the photograph of the setup are illustrated in Fig. 2. The entire system primarily comprises four core modules: the pump light source, the Raman shift cell, the photoacoustic cell, and the signal acquisition and processing module.

A pulsed Nd:YAG solid-state laser was employed as the pump source (wavelength: 532 nm; pulse width: 7 ns; repetition rate: 10 Hz; maximum single-pulse energy: 100 mJ; beam diameter: ∼6 mm). The pump beam was focused by a long-focal-length focusing lens (*f* = 50 cm) into a stainless steel Raman cell with a length of 1 m and a diameter of 20 mm. To minimize insertion loss, the Raman cell featured a specialized optical design: the input port utilized a UV fused silica window coated with a visible (VIS) anti-reflection (AR) film, while the output port was custom-designed as a long-focal-length convex lens window to seal the gas while achieving collimated output of the divergent beam.

High-purity hydrogen (99.9%) was filled into the Raman cell as the Raman-active medium. The primary rationale for selecting high-purity hydrogen is to achieve precise frequency self-matching, ensuring that the generated Stokes frequency shift strictly corresponds to the Raman transition of the target hydrogen molecules (*Q*(1) branch, ∼4155 cm⁻¹ ), thereby eliminating the need for complex wavelength tuning. Furthermore, the high-purity environment effectively prevents nonlinear gain competition and multi-wavelength cross-interference introduced by impurity gases.

As illustrated in Fig. 2(a), at the output of the Raman cell, the generated 683 nm first-order Stokes light ($\omega_{s}$) and the attenuated 532 nm pump light ($\omega_{p}$) constitute a naturally collinear dual-color excitation field. Subsequently, this dual-color light is refocused by an achromatic lens (*f* = 15 cm) into the center of the photoacoustic cell, ensuring high spatial and temporal overlap, and converged at the center of the photoacoustic cell to excite nonlinear interactions. Compared to traditional schemes employing two independent lasers for the pump and Stokes sources, this design significantly reduces system complexity and hardware costs. Furthermore, the intrinsic collinearity of the two beams output from the Raman shifter not only simplifies optical alignment but also substantially enhances the system’s anti-interference capability and stability during long-term operation. Inside the photoacoustic cell, the dual-color light field induces energy deposition (ΔU) through nonlinear interactions, exciting acoustic waves that are ultimately captured by the microphone and converted into an acoustic pressure signal (Δp) reflecting the hydrogen concentration.

Regarding photoacoustic signal generation, the core component is a differential H-type resonant photoacoustic cell. The cell adopts a symmetric structure, comprising two identical cylindrical acoustic resonant tubes and corresponding buffer chambers, sealed at both ends with VIS AR-coated UV fused silica windows. The acoustic signals generated by the stimulated Raman interaction were synchronously picked up by two high-sensitivity microphones (50 mV/Pa) installed at the midpoint of the resonant tubes.

The microphone signals were sequentially processed by a low-noise preamplifier, a bandpass filter, and a differential amplifier circuit with a high common-mode rejection ratio (CMRR). The differential detection method effectively suppresses environmental flow noise and electromagnetic interference while doubling the photoacoustic signal amplitude, thereby significantly improving the system’s signal-to-noise ratio (SNR). Finally, the analog signals were digitized by a high-speed data acquisition card (DAQ) and transmitted to a computer. The signal amplitude at the resonance frequency was extracted via Fast Fourier Transform (FFT), and the concentration of the target hydrogen gas was retrieved using a least-squares fitting algorithm.

### 3.2 Optimization and analysis of stimulated Raman scattering

Stimulated Raman scattering (SRS) serves as an effective frequency conversion technique, characterized by high conversion efficiency, superior beam quality, and high power scalability [43], [44]. Its conversion threshold is intimately correlated with the molecular number density (i.e., pressure) of the Raman medium (hydrogen), the length of the Raman cell, and the pump laser power. In this study, with the cell length and pump power fixed, the energy conversion efficiency from pump light ($I_{p}$) to Stokes light ($I_{s}$) can be modulated by optimizing the pressure within the Raman cell. According to the theoretical model in Eq. (13), the amplitude of the photoacoustic signal ($S_{\text{PARS}}$) is directly proportional to the product of the pump intensity ($I_{p}$) and the first-order Stokes intensity ($I_{s}$). Consequently, optimal photoacoustic detection sensitivity can be achieved by tuning the pressure of the Raman cell.

**Fig. 3:**
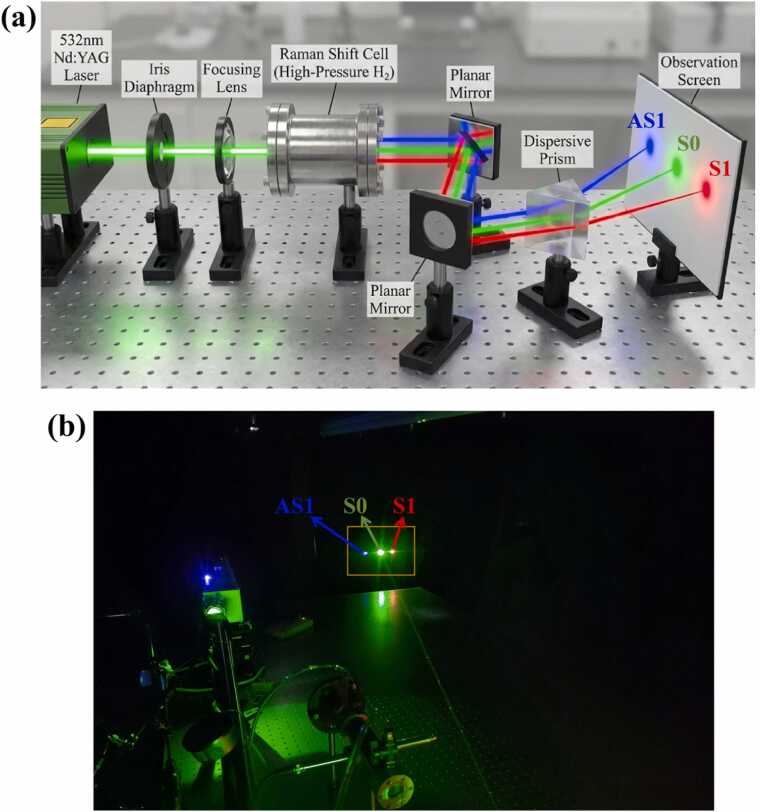
Output beam separation system of the Raman cell. (a) 3D model; (b) Experimentally observed beam splitting effect.

To systematically analyze the variation trends of the output of various Raman frequency-shifted orders under different pressures, an output beam separation system was designed. Its three-dimensional (3D) model and the experimentally observed beam splitting effect are shown in Fig. 3. An energy meter was employed to sequentially measure the beams separated by a dispersive prism, analyzing the energy variation laws of various Raman frequency-shifted components within a pressure range of 1–24 atm.

For pure gases, the concentration is primarily regulated by the filling pressure. Excessively low pressure results in insufficient molecular number density to reach the SRS threshold, whereas excessively high pressure may generate high-order Stokes light, causing energy dispersion. As the pressure varied, the Raman cell generated not only first-order Stokes light (S1, 683 nm) but also higher-order Stokes light (S2, 954 nm) and anti-Stokes light (AS1, 436 nm).

**Fig. 4:**
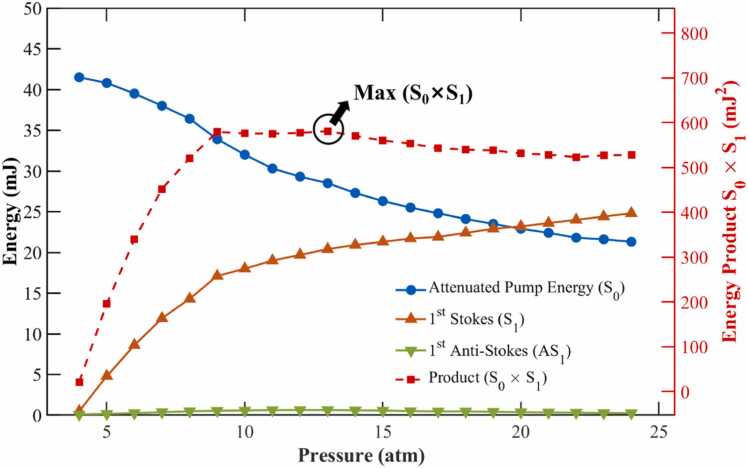
Relationship between various Raman frequency-shifted components and the internal pressure of the Raman cell.

The variations of attenuated pump light (S0), first-order Stokes light (S1), anti-Stokes light (AS1), and the raw energy product (S0×S1) with pressure are illustrated in Fig. 4. Here, S0 ×S1 reflects the interaction strength between the pump and Stokes light fields during the SRS process; the plotted values are calculated directly without normalization. To ensure the accuracy of energy measurements, the energy meter used in the experiment (NOVA II, OPHIR; PE50BF-DIF-C) was calibrated for spectral responsivity across all specific wavelength components (e.g., 532 nm, 683 nm). Simultaneously, the plotted data were corrected based on the dispersion characteristics ($n_{436}\text{≈}1.635\text{,}n_{532}\text{≈}1.625\text{,}n_{683}\text{≈}1.615$) of the N-F2 prism (LBTEK, EDP125) and Fresnel equations to eliminate the influence of wavelength-dependent surface reflection losses on the energy product calculation.

The measurement results correlating the intensity of each Raman frequency-shifted component with the internal pressure of the Raman cell are presented in Fig. 4. Since the optimization objective is to maximize the product of S0 and S1, the range of 1–4 atm, where S1 has not yet been significantly converted, is not discussed. As the pressure within the Raman cell increased, S0 exhibited a monotonically decreasing trend, with a rapid decline observed between 6 and 10 atm. In this range, the increased pressure enhanced the Raman gain, causing a substantial conversion of pump light into S1. With further pressure increases, the growth of the S1 effect slowed, and the attenuation of S0 correspondingly decelerated. The intensity of the S1 component initially showed a rapid exponential growth trend in the low-pressure region, primarily because the increased number density of hydrogen molecules directly enhanced the steady-state Raman gain coefficient. However, as the pressure rose above 10 atm, the growth of S1 gradually tapered off, showing a tendency to enter a saturation region. This phenomenon is attributed to the collisional broadening effect at high pressures, which increases the Raman linewidth [45], thereby partially offsetting the gain enhancement brought by the increased density. Simultaneously, the rapid depletion of the pump light also limited the growth rate of the S1 energy.

The overall energy of the AS1 component was relatively low, exhibiting non-monotonic variation and reaching a peak at 13 atm. This phenomenon reveals the dominant role of the Four-Wave Mixing (FWM) effect [46]. The generation of AS1 relies primarily on this FWM parametric process, which must satisfy a strict phase-matching condition ($\text{Δ}k=2k_{p}-k_{s}-k_{\mathrm{as}}\approx 0$). The dispersion characteristics of the gas change with pressure, rendering the FWM efficiency highly sensitive to pressure variations. Around 13 atm, the dispersion properties of hydrogen result in Δk≈0, satisfying the optimal phase-matching condition. This maximizes the coherence length, enabling efficient energy transfer to the anti-Stokes component. As the pressure continues to rise beyond 13 atm, dispersion causes the phase mismatch to increase again, leading to the attenuation of the AS1 signal intensity.

The product S0 ×S1 presented a trend of initially rising and then falling, also reaching its peak at 13 atm. The upward trend of S0 ×S1 in the low-pressure region was triggered by the rapid exponential growth of S1. When the optimal balance point was reached, the energy of S1 was sufficiently strong while the pump light had not yet been excessively depleted. At this moment, S0 ×S1 reached its maximum. As the pressure increased further, although S1 continued to rise slowly or maintained a high level, the significant decline in pump light dominated the trend of the product, causing the total product to decrease.

It is noteworthy that the pressure corresponding to the peak of S0 ×S1 coincides with that of the AS1 peak. This is not a coincidence, but rather a manifestation of the synergistic collaboration and collective optimization of multiple nonlinear processes, including stimulated Raman scattering and four-wave mixing. The generation efficiency of FWM depends not only on phase matching but also strongly on the energy intensity of the nonlinearly interacting optical fields. This fully demonstrates that 13 atm is the optimal equilibrium point for energy conversion, endowing the system with both the strongest interacting optical fields and the optimal phase-matching environment simultaneously, thereby maximizing the generation efficiency of AS1.

To accurately assess the energy distribution, we conducted a dedicated measurement of the second-order Stokes wave (S2, 954 nm). At the optimal pressure of 13 atm, although a faint S2 signal was qualitatively observed using an infrared detection card, its measured pulse energy was only ∼0.29 mJ, corresponding to approximately 1.5% of the S1 energy (∼19.7 mJ). This indicates that the pump depletion induced by the cascade effect is negligible at this pressure level. Consequently, the analysis in this study focuses primarily on the dominant S0 and S1 components.

Considering the conversion characteristics of S0 ×S1 and the experimental analysis of the optimal phase-matching point for AS1, 13 atm was finally determined as the optimal working pressure. At this pressure, the 532 nm S0 and the 683 nm S1 propagate collinearly, providing the optimal excitation conditions for subsequent high-sensitivity PARS detection.

### 3.3 Simulation optimization and analysis of photoacoustic cell acoustic field

As the venue for photoacoustic interaction and the core device for acoustic signal resonance enhancement, the photoacoustic cell (PAC) possesses acoustic characteristics that directly determine the detection sensitivity and signal-to-noise ratio (SNR) of the entire system [47], [48]. To achieve high-sensitivity detection, a targeted dual noise reduction strategy was adopted. First, addressing the coherent photothermal noise from the windows generated by high-power pulsed laser bombardment (which excites the anti-symmetric acoustic mode and thus cannot be eliminated by electronic differentiation), buffer volumes were designed at both ends of the resonant tubes to provide physical attenuation. Second, a differential H-type structure with symmetric dual-microphone detection was employed to efficiently suppress random common-mode noise such as flow turbulence, mechanical vibration, and electromagnetic interference.

Structurally, high-sensitivity microphones are coupled and mounted at the axial midpoints of the two resonant tubes, (specifically, the antinodes of the acoustic pressure standing wave), to capture the maximum sound pressure response. When the excitation beam passes through one of the resonant tubes, the acoustic coupling effect of the H-type structure excites a specific longitudinal resonant mode within both tubes, characterized by equal amplitudes and opposite phases (i.e., a 180°phase difference). In contrast, external random interferences typically manifest as in-phase common-mode signals. Consequently, employing differential signal processing technology not only effectively cancels out common-mode noise but also theoretically doubles the amplitude of the effective photoacoustic signal, thereby significantly enhancing the system’s SNR.

**Fig. 5:**
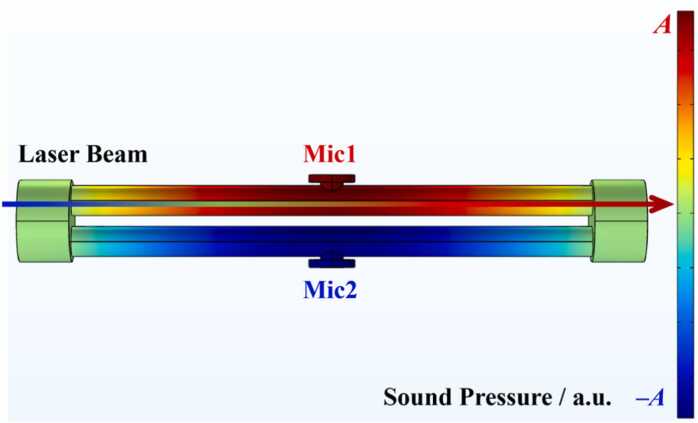
Acoustic field distribution within the photoacoustic cell.

To validate the rationality of the differential resonant tube design and further optimize its acoustic performance, numerical simulations of the internal acoustic field distribution were conducted using the thermoviscous acoustics module in finite element analysis software. The analysis focused on the first-order longitudinal resonant mode. Although the pressure of the Raman cell was optimized to 13 atm in Section 3.2 to maximize the optical field conversion efficiency, the photoacoustic cell, as the detection unit, maintains a standard atmospheric pressure (1 atm) environment throughout the measurement process. Therefore, in the simulation settings, the fluid domain within the photoacoustic cell was defined as standard dry air at 1 atm. Furthermore, according to the ideal gas law, the speed of sound ($v\approx \sqrt{\gamma \mathrm{RT}/M}$) depends primarily on gas composition under constant temperature control and is minimally affected by pressure. This implies that the acoustic resonance mode distribution of the photoacoustic cell exhibits high consistency between 1 atm and theoretical high-pressure conditions. All wall surfaces were set as hard sound boundary conditions to simulate the high acoustic reflectivity of the cell’s metallic inner walls. A modal analysis of the acoustic field distribution was performed using an eigenfrequency solver, with the results presented in Fig. 5.

At the axial center positions of the two acoustic resonant tubes, the sound pressure amplitude reaches its maximum (antinode). More critically, the sound pressure signals within the two resonant tubes exhibit strictly anti-phase characteristics (a phase difference of 180°), validating the aforementioned differential design theory.

**Fig. 6:**
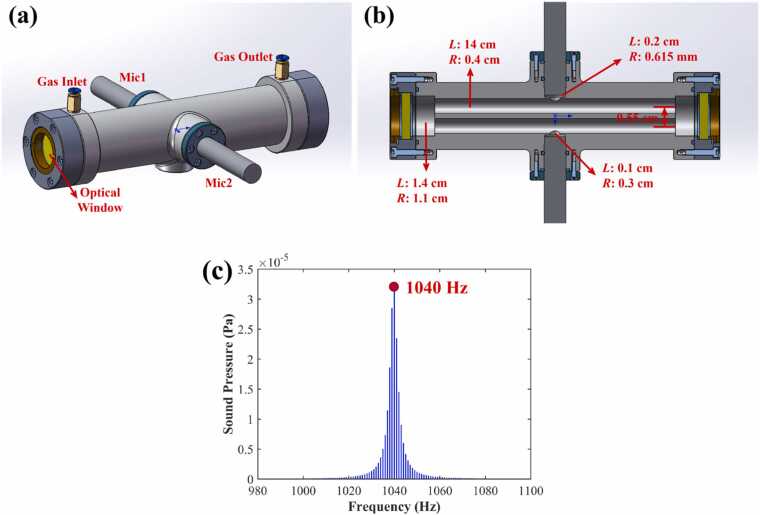
Optimization results of photoacoustic cell parameters. (a) 3D model; (b) Internal parameters of the photoacoustic cell; (c) Sound-Frequency characteristics curve.

Furthermore, the geometric parameters of the photoacoustic cell have a decisive impact on its resonance frequency, quality factor (*Q*-factor), and the ultimate photoacoustic signal intensity. To achieve optimal acoustic resonance enhancement at a specific frequency while effectively suppressing background noise, the acoustic frequency characteristics were optimized by adjusting the length and radius of the resonant tubes, the length and radius of the buffer chambers, the offset length of the resonant tubes from the buffer chamber center, the length and radius of the acoustic signal extraction tubes, and the height of the microphone installation gap. This iterative optimization aimed to determine the optimal set of geometric dimensions. The core objective was to maximize the sound pressure amplitude at the microphone position, subject to the constraint that the resonance frequency should be no lower than 1 kHz (to avoid low-frequency environmental noise) and not excessively high (to prevent rapid signal attenuation). The final optimized parameters are shown in Fig. 6. The simulated resonance frequency of the photoacoustic cell is approximately 1040 Hz.

To quantify the noise suppression capability of the differential configuration in practice, we evaluated the Common-Mode Rejection Ratio (CMRR) using an external acoustic source. A piezoelectric buzzer was placed at the gas inlet to generate common-mode noise at the resonance frequency (1110 Hz), representing the worst-case scenario for acoustic symmetry. The measured single-ended signal amplitude was 1.344 (a.u)., while the differential signal amplitude was suppressed to 0.175 (a.u.). Based on the formula $\text{CMRR}=20\log(V_{\mathrm{single}}/V_{\mathrm{diff}})$, the calculated full-chain CMRR is approximately 17.7 dB. This result confirms that the differential design effectively attenuates environmental acoustic interference and flow noise by a factor of nearly 8, even considering practical limitations such as microphone mismatch and acoustic path asymmetry.

### 3.4 Photoacoustic signal processing and analysis

**Fig. 7:**
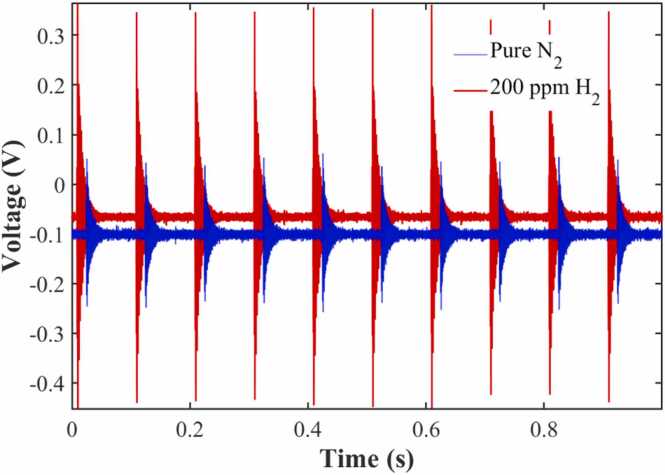
Comparison of time-domain photoacoustic signals between pure nitrogen background and 200 ppm hydrogen.

Precise calibration of the photoacoustic cell's resonance frequency and confirmation of the signal source are prerequisites for achieving high-sensitivity detection. To verify that the detected signal originates from the SRS effect of hydrogen rather than purely from window photothermal noise, a comparative test of time-domain signals for pure nitrogen and 200 ppm hydrogen was conducted, as shown in Fig. 7 (laser repetition rate: 10 Hz). Both the pure nitrogen background (blue curve) and the 200 ppm hydrogen signal (red curve) exhibit similar 10 Hz impulsive features in the time domain, confirming that high-energy pulsed laser bombardment on the windows indeed generates photothermal effects. However, a comparison reveals that the overall signal amplitude of 200 ppm hydrogen is significantly higher than that of the pure nitrogen background, and it exhibits a more distinct damped acoustic oscillation following the pulse excitation. Furthermore, the slight vertical deviation in the baselines of the two raw signals primarily stems from DC drift in the microphone pre-amplifier circuit. Since this DC component carries no acoustic frequency information, it is completely eliminated after subsequent digital bandpass filtering and does not affect the extraction and analysis of the AC photoacoustic signal.

The excitation mechanism of this system differs fundamentally from Continuous Wave (CW) photoacoustic techniques. While CW techniques require the laser modulation frequency to be strictly locked to the acoustic resonance frequency, this system utilizes a nanosecond pulsed laser for impulse excitation. The 7 ns laser pulse manifests as a broadband acoustic source in the frequency domain, with its spectral energy naturally covering the first-order longitudinal resonance frequency of the photoacoustic cell (∼1110 Hz). Consequently, upon excitation, the photoacoustic cell generates a damped oscillation (i.e., ringing signal) at its eigenfrequency. The laser repetition rate (10 Hz) determines only the data sampling rate and does not need to match the acoustic resonance frequency.

It is worth noting that the adoption of a 10 Hz low-repetition-rate pulsed excitation scheme in this system is primarily based on the nonlinear physical mechanism of SRS and the optimization trade-off for the signal-to-noise ratio (SNR). First, Stimulated Raman Scattering (SRS) is a third-order nonlinear optical process, the occurrence of which strictly depends on an extremely high peak power density (typically on the order of MW/cm²) to overcome the nonlinear threshold. The nanosecond pulsed laser used in this system provides MW-level peak power, which is approximately 6 orders of magnitude higher than that of Watt-level (W) CW lasers. This immense nonlinear gain advantage far exceeds the linear acoustic resonance accumulation provided by CW modulation (which typically depends on the quality factor Q, roughly a few tens). Second, regarding the SNR trade-off, the low repetition rate of 10 Hz provides a thermal relaxation time of approximately 100 ms, effectively avoiding window heat accumulation and baseline drift issues common in high-repetition-rate or CW schemes. Therefore, this scheme ensures efficient SRS excitation while maintaining background noise at a minimum level, representing the optimal engineering balance for achieving high-sensitivity detection. Furthermore, regarding the optimization of excitation intensity, the product of S0 and S1 energies was maximized by adjusting the Raman cell pressure (see Section 3.2), thereby ensuring optimal signal excitation efficiency.

Due to the weak amplitude of the effective photoacoustic signal and the presence of strong background noise and electromagnetic interference, hardware-level pre-processing was implemented. Specifically, a dedicated low-noise pre-amplifier circuit was designed with a bandpass filter set to 500–1500 Hz to cover the predicted resonance frequency range and eliminate low-frequency flow noise. However, the Signal-to-Noise Ratio (SNR) of the time-domain photoacoustic signal remains low, making it difficult to perform accurate quantitative analysis of the signal intensity based solely on voltage amplitude features.

**Fig. 8:**
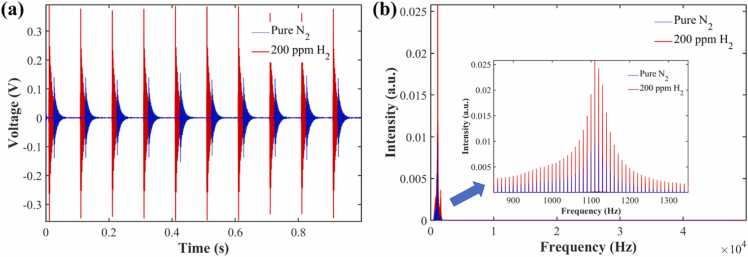
Software algorithm processing results of photoacoustic signals. (a) Digital filtering; (b) FFT spectrum conversion.

To address this low SNR challenge, a high-precision digital signal processing algorithm was designed to further optimize the raw acquired data, building upon the hardware noise reduction. First, to maximally filter out broadband random noise without compromising the effective signal, a 4th-order Butterworth digital bandpass filter was employed to process the time-domain signal. Compared to conventional filtering algorithms, the Butterworth filter exhibits a maximally flat frequency response within the passband, ensuring no distortion in the amplitude of the photoacoustic signal. The filter passband was set to 500–1500 Hz. The processed time-domain waveform is shown in Fig. 8(a), where it is evident that the baseline noise has been significantly suppressed.

Subsequently, to precisely extract the signal amplitude at the resonance frequency, a frequency-domain analysis was performed on the filtered data. Considering that the truncation of finite-length data induces spectral leakage, which affects the accuracy of peak extraction, a Hanning window function was introduced to weight the time-domain signal prior to executing the Fast Fourier Transform (FFT). This window function effectively suppresses sidelobes and enhances spectral resolution. Furthermore, an amplitude correction algorithm was applied to compensate for the energy loss caused by windowing, thereby recovering the true spectral distribution.

As shown in Fig. 8(b), the spectrum processed via the windowed FFT exhibits a sharp spectral peak with an extremely high SNR at 1110 Hz. Comparing the frequency-domain amplitudes, the noise amplitude of the pure nitrogen background at the 1110 Hz resonance frequency is only 0.009487 (a.u.). Under the same experimental conditions, upon filling with 200 ppm hydrogen, the resonance signal amplitude at this frequency significantly increases to 0.0258 (a.u.). This signal enhancement of approximately 2.72 times powerfully demonstrates that, despite the existence of certain window background noise in the system, the detected 1110 Hz resonance energy is dominated by the stimulated Raman relaxation process of the hydrogen molecules. This precisely identified the first-order longitudinal resonance frequency of the photoacoustic cell (which is consistent with the simulated value of 1040 Hz; the minor deviation is primarily attributed to machining errors and the idealized simplifications in the simulation model).

To further verify the accuracy of the simulation model, we calculated and compared the simulated normalized frequency response curve with the experimental spectrum. The results show that although the quality factor predicted by simulation (Q≈247) is higher than the experimental value (Q≈28), this is primarily attributed to the acoustic loading effect of the microphone diaphragm, microscopic roughness of the inner walls, and others. These factors not fully accounted for in the idealized hard-boundary simulation. This phenomenon of Q value deviation is relatively common in photoacoustic cell research and does not affect the simulation model's accurate prediction of acoustic modes and structural optimization trends [6].

Additionally, the background noise was suppressed to an extremely low level. These results validate the effectiveness of the combined “Butterworth filtering + Hanning window FFT” algorithm in extracting weak photoacoustic signals. Consequently, the intensity value corresponding to 1110 Hz was used as the representative photoacoustic signal value for subsequent hydrogen concentration retrieval. The currently adopted frequency-domain extraction algorithm achieves a favorable balance between computational efficiency and detection sensitivity. Physically, the photoacoustic signal generated by this system manifests as a transient damped oscillation wave with a highly deterministic carrier frequency (∼1110 Hz). Although the microphone captures broadband signals containing flow noise and electromagnetic interference, the target hydrogen signal exhibits extremely distinct frequency-domain characteristics. Therefore, the combination of Butterworth band-pass filtering + Hanning windowed FFT effectively constructs an efficient digital narrowband filter, which can precisely lock onto and extract the resonance peak energy from broadband background noise with minimal computational cost. In comparison, while time-frequency analysis algorithms such as Wavelet Transform possess advantages in processing non-stationary transient signals, for the fixed-frequency signals in this study, the improvement in signal-to-noise ratio (SNR) is not significant compared to the finely optimized FFT, and the computational complexity is considerably higher. Future work will focus on exploring intelligent denoising networks based on deep learning, utilizing data-driven approaches to further suppress nonlinear background noise and enhance the system's detection performance.

### 3.5 Hydrogen detection experiments and analysis

Upon precisely locking the first-order longitudinal resonance frequency of the photoacoustic cell, gas concentration calibration experiments were conducted to quantitatively evaluate the linear response characteristics of the system. A high-precision Mass Flow Controller (MFC) was used to prepare 9 sets of standard hydrogen samples with varying concentrations (0, 10, 50, 100, 200, 300, 500, 750, 1000 ppm). The amplitudes of the photoacoustic signals for each concentration were recorded at the resonance frequency. Each data point represents the average of 10 independent measurements, with an integration time of 5 s per measurement. The standard deviation is plotted as error bars in the results to reflect the system's repeatability and stability. Considering that gas temperature and pressure directly affect the speed of sound and the resonance characteristics, a precision heating film was attached to the cell wall, and the internal gas temperature was maintained at 25℃ via a feedback control system. All concentration calibration experiments were conducted under standard atmospheric pressure (1 atm).

**Fig. 9:**
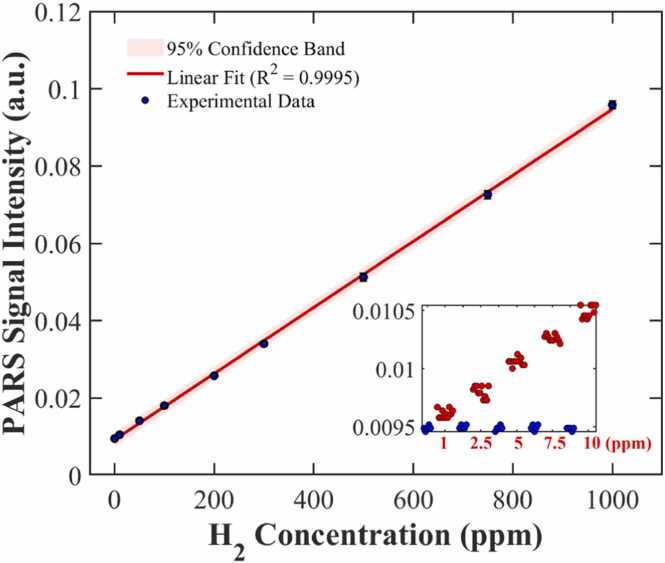
Hydrogen detection results based on the DPA-SRS system.

Fig. 9 illustrates the fitting relationship between the photoacoustic signal amplitude and hydrogen concentration, along with the 95% confidence bands. A linear regression analysis performed on the experimental data using the least squares method demonstrates that the system exhibits excellent linearity, with the coefficient of determination (*R*^2^) reaching 0*.9995.* The calculated fitting slope (sensitivity k) is $8.5461\text{×}10^{-5}\text{ V}/\text{ppm}$, with a 95% Confidence Interval (CI) of $\text{[}8.370\text{×}10^{-5}\text{,}8.722\text{×}10^{-5}]$; the linear intercept (b) is 0.0092133, with a 95% CI of [0.0084, 0.0100]. The extremely narrow confidence interval range (slope error only approx. ±2%) further confirms the statistical reliability of the system’s linear response.

Within the current measurement range, the system demonstrates excellent linear response. However, if the hydrogen concentration increases significantly (e.g., to percentage levels), pump depletion may occur during the stimulated Raman process. Furthermore, the decrease in the average molecular weight of the gas mixture would lead to an increase in the speed of sound, subsequently causing an upward drift in the resonance frequency of the photoacoustic cell. According to the sound speed calculation model, when the hydrogen concentration is 1000 ppm, the induced frequency shift is extremely marginal (approximately 0.5 Hz); thus, its impact on the current quantitative accuracy is negligible.

To further investigate the ultimate detection sensitivity of the system, a set of trace hydrogen standard samples with graded concentrations (1, 2.5, 5, 7.5, and 10.0 ppm) was specifically prepared. These low-concentration samples were tested sequentially, the results are shown in the Inset of Fig. 9. Based on the standard deviation of 25 groups of background noise measured in a pure nitrogen environment ($\sigma \text{=}1.85\text{×}10^{-5}\text{ V}$), the theoretical Limit of Detection (LOD) calculated according to the 3σ criterion [6] is approximately 0.65 ppm. This theoretical value aligns highly with the distinct step-like experimental signals observed in the low-concentration region (0–10 ppm) shown in the inset, demonstrating that the system possesses solid detection capability and high SNR even at extremely low concentrations.

It should be noted that the high-energy nanosecond pulsed excitation (100 mJ, 7 ns) employed in this system induces significant thermal stress accumulation on the optical windows. To ensure that the optical components operate within their damage thresholds and to minimize nonlinear thermal interference, this study adopted a 5 s burst sampling mode (totaling 50 pulses). Constrained by the physical characteristics of the pulsed light source and the adsorption effects of the aluminum cell body, evaluating the LOD using the standard deviation of background noise fluctuations (3σ criterion) represents the method that most purely reflects the intrinsic sensitivity of the stimulated Raman differential excitation mechanism at this stage. This evaluation logic is consistent with the aforementioned literature [6], [22]. Future work will further explore the system's stability limits by developing novel photoacoustic cells using passivated stainless steel and spatial filtering designs.

### 3.6 Comparison with advanced technologies

To comprehensively evaluate the overall performance of the DPA-SRS system, a multi-dimensional benchmark comparison was conducted against frontier hydrogen detection technologies reported in recent years, covering key indicators such as detection limit, response speed, and system complexity. The results are presented in Table 1.

**Table 1:** Comparison of key performance indicators between the proposed DPA-SRS system and other advanced hydrogen detection methods.

**Technique**	**Detection Principle**	**LOD (ppm)**	**Response Time (s)**	**Excitation Source**	**System Complexity**	**Limitations**	**Refer-ence**
DPA-SRS	Stimulated Raman + Acoustic	0.6	5	532 nm Pulsed (100 mJ)	Medium	High peak power laser required	This work
CERS (Cavity-enhanced)	Spontaneous Raman	50	20	532 nm CW (1.5 W)	High (Precision alignment)	Alignment sensitive;	[36]
MNF-SRS	Stimulated Raman	122	6	1532 nm CW (1 W)	High (Dual lasers + EDFA)	Complex setup	[49]
Gas Chromatography	Separation + Ionization	0.01	> 600	N/A (Carrier Gas)	High (Valves + Carrier gas)	Requires consumables	[50]
PAS (Frequency Shift)	Acoustic Resonance Shift	139	0.5	1368 nm Modulated DFB	Low (Single diode)	Needs pump gas	[28]
Pd/3D-Graphene	Chemiresis -tive	1000	8	N/A (Voltage)	Low (Chip-scale)	Humidity interference	[51]

Gas Chromatography (GC) represents the current gold standard for sensitivity, with portable systems achieving detection limits as low as ∼10 ppb [50]. However, limited by the chromatographic column separation mechanism, its analysis cycle exceeds 10 min and relies on consumable carrier gas, making it difficult to meet the demand for real-time early warning of transient leakage events in industrial sites. Resistive sensors (e.g., Pd/3D-graphene [51]), while offering rapid response, are limited by relatively high detection limits (> 1000 ppm) and environmental humidity interference. In contrast, the proposed system achieves a detection limit of 0.6 ppm while maintaining a 5 s real-time response, effectively resolving the trade-off between high sensitivity and high timeliness.

In the field of optical detection, the latest Cavity-Enhanced Raman Spectroscopy (CERS) [36], despite utilizing a 1.5 W high-power continuous-wave laser, remains limited to a detection limit of ∼50 ppm. Similarly, Micro-Nano Fiber Stimulated Raman Spectroscopy (MNF-SRS) [49] is limited by weak evanescent field interaction, achieving a detection limit of only ∼122 ppm. By leveraging high-energy pulses to induce intense stimulated Raman gain in free space, combined with acoustic resonance enhancement, DPA-SRS achieves a sensitivity improvement of two orders of magnitude compared to indirect Photoacoustic Spectroscopy (PAS) [28].

Beyond performance indicators, engineering applicability is crucial for field monitoring. CERS [36] and MNF-SRS [49] rely on alignment-sensitive multi-pass optical cavities and complex 'dual-laser + fiber amplifier' configurations, respectively; minor mechanical vibrations or temperature drifts can lead to cavity detuning or significant signal fluctuations. GC [50] involves complex mechanical valve switching and heating cycles. In comparison, DPA-SRS adopts a more robust single-path free-space excitation scheme. It requires neither precise cavity mode matching nor a second probe laser. Although the current integration level of nanosecond pulsed sources is lower than that of diode lasers, with the development of miniaturized solid-state laser technology, DPA-SRS provides superior long-term stability and engineering robustness compared to precision optical cavity technologies while ensuring ultra-high sensitivity.

## 4 Conclusion

This study investigated a hydrogen detection system based on SRS enhancement combined with differential resonant photoacoustic technology. By achieving deep coupling and synergistic optimization between nonlinear excitation optical field modulation and high-sensitivity acoustic detection mechanisms, the system effectively addresses the challenge of trace detection of non-polar hydrogen molecules at ambient temperature and pressure.

In terms of optical excitation, the nonlinear dynamic processes in high-pressure hydrogen were systematically analyzed, revealing the synergy mechanisms between SRS gain and Four-Wave Mixing (FWM) effects. A pressure of 13 atm was identified as the optimal balance point for the conversion rate of pump light to first-order Stokes light, thereby successfully constructing an efficient dual-wavelength excitation optical field based on the synergistic regulation of nonlinear effects. Secondly, regarding acoustic signal detection, a differential H-type resonant photoacoustic cell was developed. By leveraging the characteristics of the first-order longitudinal resonant mode, coherent enhancement of the photoacoustic signal and differential suppression of background common-mode noise were realized, significantly improving the system’s signal-to-noise ratio (SNR). In terms of acoustic signal analysis, by combining hardware and software filtering with weak signal extraction algorithms, the precise analysis of photoacoustic signals was achieved.

Additionally, this system achieves adaptive dual-wavelength excitation without the need for wavelength tuning using only a single fixed-frequency laser, effectively circumventing the reliance on complex tuning systems inherent in traditional dual-laser dual-wavelength detection methods. Experimental results demonstrate that under standard environmental conditions, the system achieved effective extraction of hydrogen photoacoustic signals at 1 ppm and a limit of detection (LOD) of 0.65 ppm (3*σ*), while maintaining excellent linearity (*R*^2^ > 0.999) across a wide concentration range. This research provides a feasible and robust spectroscopic solution for trace non-polar gas sensing in industrial and safety fields.

## CRediT authorship contribution statement

**Jiaxiang Liu:** Data curation. **Zhengang Li:** Writing – review & editing, Resources, Methodology, Investigation, Funding acquisition, Conceptualization. **Xin Yu:** Writing – original draft, Visualization, Software, Methodology, Formal analysis. **Yonghua Fang:** Supervision, Project administration, Methodology, Funding acquisition. **Ying Pan:** Resources. **Yongqing Fang:** Visualization. **Canlong Wang:** Resources. **Junfang Miao:** Validation, Software. **Haichun Xu:** Visualization, Formal analysis.

## Declaration of Competing Interest

The authors declare that they have no known competing financial interests or personal relationships that could have appeared to influence the work reported in this paper.

## Data Availability

Data will be made available on request.
